# Nationwide epidemiological analysis of surgically treated upper limb vascular trauma over 16 years in Brazil

**DOI:** 10.31744/einstein_journal/2025AO1339

**Published:** 2025-10-03

**Authors:** Marcella Moura Ceratti, Carolina Carvalho Jansen Sorbello, Felipe Soares Oliveira Portela, Marcelo Fiorelli Alexandrino da Silva, Marcelo Passos Teivelis, Nelson Wolosker

**Affiliations:** 1 Hospital Israelita Albert Einstein São Paulo SP Brazil Hospital Israelita Albert Einstein, São Paulo, SP, Brazil.; 2 Universidade de São Paulo Faculdade de Medicina Department of Vascular and Endovascular Surgery São Paulo SP Brazil Department of Vascular and Endovascular Surgery, Faculdade de Medicina, Universidade de São Paulo, São Paulo, SP, Brazil.; 3 Hospital Israelita Albert Einstein Faculdade Israelita de Ciências da Saúde Albert Einstein São Paulo SP Brazil Faculdade Israelita de Ciências da Saúde Albert Einstein, Hospital Israelita Albert Einstein, São Paulo, SP, Brazil.

**Keywords:** Vascular injury, Upper extremity, Health Information System, Epidemiology, Incidence, Data mining, Brazil

## Abstract

**Objective:**

To evaluate the epidemiology of upper limb vascular trauma across Brazil and evaluate the incidence rates, demographic characteristics, lethality, days of hospitalization, and related healthcare costs.

**Methods:**

This retrospective analysis used data from the Brazilian public health system over sixteen years (2008–2023). Automated data extraction utilized Python-based tools to gather information on vascular trauma procedures identified by ICD-10 codes. Statistical analyses were performed to assess variations in incidence, lethality, and treatment costs across Brazilian regions.

**Results:**

A total of 25,573 cases of upper limb vascular trauma were recorded. Most cases occurred in males (79.8%) with a mean age of 34.7 years. The region in Brazil with the highest incidence of upper limb vascular trauma was the North (16.6 cases per 100,000 inhabitants), while the Southeast had the lowest (10.7 cases per 100,000 inhabitants). The average length of hospital stay was 4.39 days, and 92.8% of the patients did not require admission to the intensive care unit. Among patients admitted to the intensive care unit, the average length of stay was 4.52 days. The overall lethality of upper limb vascular trauma was 2.37%, with higher lethality observed in bilateral upper limb vascular trauma (3.81%).

**Conclusion:**

The incidence of upper limb vascular trauma is higher in Brazil than in developed countries, even after adjusting for population size. However, lethality and hospitalization duration did not appear to differ significantly from those in developed countries.

## Highlights

■Brazil had a higher upper limb vascular trauma incidence than the US, remaining stable over the years.

■The region with the highest proportion of cases was the North, followed by the Southeast.

■The most affected population was young men, with a lethality of 2.37%, comparable to that globally.

■Most patients did not require intensive care (92.8%), and the average hospital stay was 4.39 days.

## In Brief

This 16-year nationwide study analyzed patients with surgically treated upper limb vascular trauma in Brazil. The findings show that upper limb vascular trauma incidence was higher than that in developed countries; however, the lethality and hospital stays are comparable. This condition was more common in young males. These results highlight the burden and need for targeted trauma prevention policies.

## Graphical abstract

**Figure:**
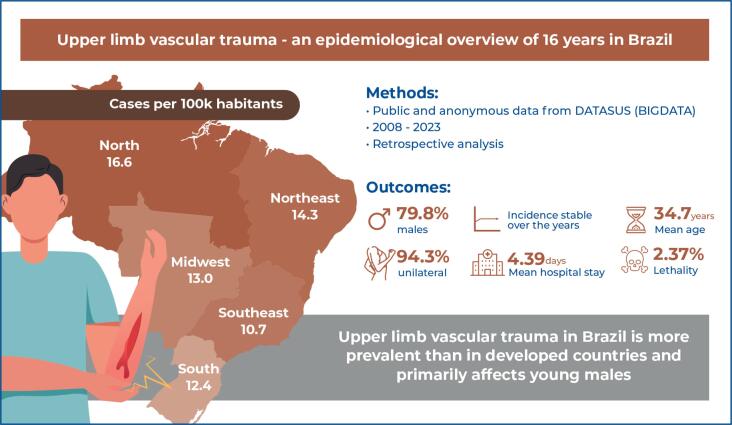

## INTRODUCTION

Vascular trauma accounts for approximately 2% of all trauma cases in developed countries,^(1)^ leading to increased morbidity and mortality. It can result in severe bleeding, ischemic limb loss, and even death,^(2)^ underscoring the need for awareness of this condition within the medical community.

Upper limb vascular trauma (ULVT) constitutes a significant proportion of vascular injuries, accounting for 26% of vascular traumas in the United States,^(1)^ 23% in Scotland,^(3)^ 64% in Australia,^(4)^ and 21% in New Zealand.^(5)^ Common causes of ULVT include accidents involving sharp objects, contusions, machinery accidents, and fractures.^(6)^

Despite the significance of ULVT, few epidemiological studies based on large population data in the literature have analyzed the impact of these injuries on health systems. Only six major studies were identified from four developed nations, including the United States, Australia, Scotland, and New Zealand. The mortality rate of patients with vascular trauma was approximately 20% in the United States^(1,6)^ and 3.6% in New Zealand. Among patients with ULVT, the lethality was 9% in Scotland^(3)^ and 2.9% in the United States.^(1)^

Despite the relevance of these studies, all data were obtained from developed countries and cannot be extrapolated to other regions. Therefore, there is limited literature regarding the epidemiology of vascular limb trauma in large populations, particularly in developing countries where there is a higher incidence of traffic accidents and violence.^(7)^ Furthermore, no large-scale study has assessed the healthcare cost of ULVT.

Brazil had a population of 203,080,756 individuals in 2022.^(8)^ In Brazil, anyone with a health complaint can access care through the Brazilian Public Health System (SUS - *Sistema Único de Saúde*); however, only 71% of the population relies on SUS exclusively.^(9)^ The SUS is considered the largest in the world, as it serves more than 200 million people.^(10)^ Public health data in Brazil, including information about surgical procedures performed by SUS, are centralized in a portal managed by the Department of Information and Informatics of the SUS (DATASUS - *Departamento de Informação e Informática do SUS*).^(11)^ This system, overseen by the Secretariat of Digital Health and Information, encompasses various health-related data, including health indicators, healthcare services, epidemiological and morbidity statistics, information on the healthcare network, vital statistics, demographic and socioeconomic data, financial details, and public health expenditures. The data were anonymized and accessed via the online DATASUS platform, which was the foundation for data collection in this study.

This report presents regional data on the incidence of ULVT in Brazil, along with demographic information (biological sex and age group), lethality, and hospitalization data, including length of hospital stay, intensive care unit admissions, and healthcare system cost.

## OBJECTIVE

This study aimed to evaluate the epidemiology of upper limb vascular trauma across Brazil, the largest country in South America and a developing nation.

## METHODS

### Study period, data source, and population

In this cross-sectional, retrospective study, data were collected from the DATASUS. This retrospective analysis focused on procedures performed for upper limb vascular trauma between 2008 and 2023. The average Brazilian population between 2008 and 2022 was approximately 196,918,278.^(8)^ Since 71% of the population exclusively uses the Brazilian SUS,^(9)^ the sample population for this study was at least 140,000,000 patients. However, because many trauma cases are initially treated in the SUS, the study population is likely to be larger (between 140 and 200 million people).

### Ethical approval

This study was approved by the Research Ethics Committee of the *Hospital Israelita Albert Einstein* (CAAE: 35826320.2.0000.0071; # 4.321.508). DATASUS serves as a public repository of anonymous data, eliminating the need for informed consent.

### Automated data extraction process

The information-gathering process was automated using a Python-based (v. 2.7.13; Beaverton, OR, USA) extraction protocol devised by the institution's IT department. Data segregation and refinement tasks were executed on a Windows 10 system using the Selenium WebDriver (v. 3.1.8; Selenium HQ) and Pandas (v. 2.7.13; Lambda Foundry, Inc. and PyData Development Team, NY, USA) tools on the DATASUS platform.

### Procedure selection on the platform

First, we selected data on trauma based on ICD-10 codes (S00-S99 and T00-T98). Subsequently, we identified patients who underwent "upper limb revascularization," "surgical treatment of bilateral upper limb traumatic vascular injuries," and "surgical treatment of unilateral upper limb traumatic vascular injuries," using DATASUS codes 0406020426, 0406020523, and 0406020531, respectively. Therefore, only data on vascular injuries to the upper limbs resulting from trauma were extracted. In this study, non-surgical ULVT was not considered. Patients with extensive tissue damage or irreversible ischemia who underwent amputation were also excluded. Notably, selection bias may have occurred because the data were obtained from records made by doctors throughout Brazil, who depended on their personal interpretations and classifications of the cases.

The extracted data provided information on the number of annual medical procedures, categorized by region, patient demographics (age and biological sex), length of hospital stay, duration in the intensive care unit (ICU), lethality, and financial reimbursements. Monetary values in Brazilian Reais (R$) were converted into U.S. dollars (US$) using the median exchange rate from 2008 to 2023, which was US$1=R$ 3.2018.^(12)^

### Data compilation and statistical analysis

Data collation and structuring were performed in.cvs format, with Microsoft Office Excel 2019 (Redmond, WA, USA) used for tabular arrangements. Population data by *age group was extracted from the Brazilian Institute of Geography and Statistics (IBGE - Instituto Brasileiro de Geografia e Estatística*).^(8)^ For statistical analysis, we used the population dependent exclusively on the Brazilian public health system, which corresponds to 71% of the total population (approximately 140 million people) according to the National Health Agency (ANS).^(9)^

Statistical analysis was performed using SPSS (version 20.0 for Windows; IBM Corp., Armonk, NY, USA). Linear regression was used to examine the progression of ULVT across the different age groups. The χ^2^ and likelihood ratio tests were employed to assess variations in ULVT rates among different macro-regions of the country, and mortality rates within regions. A significance level of p≤0.05 was deemed statistically significant for all analyses.

## RESULTS

**Figure 1:**
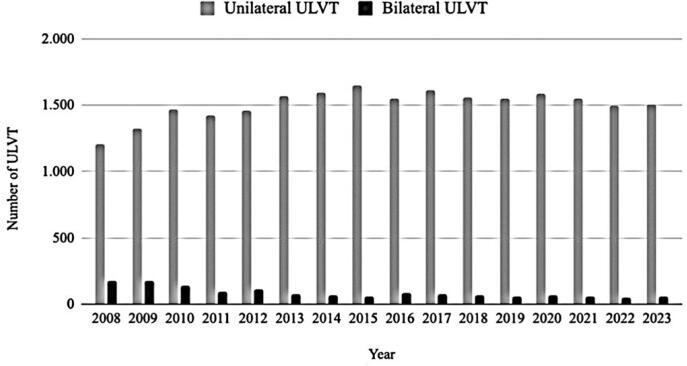
Number of upper limb vascular trauma per year in Brazil

From 2008 to 2023, there were 25,573 ULVTs. Of these cases, 24,124 (94.3%) had unilateral vascular trauma and 1,392 (5.7%) had bilateral trauma. Figure 1 shows the annual number of ULVTs in Brazil. No significant variations were observed in the incidence of ULVTs over time. Unilateral ULVT slightly increased, whereas the number of bilateral ULVT cases slightly decreased from 2008 to 2023.

ULVT: upper limb vascular trauma.

**Figure 2:**
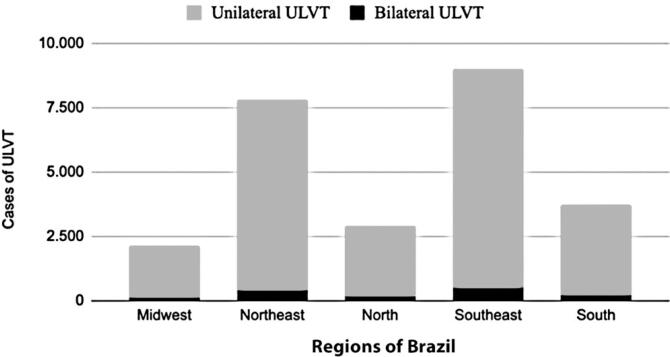
Number of upper limb vascular trauma per region of Brazil

Figure 2 shows the distribution of ULVT across five Brazilian regions. The majority of cases occurred in the Southeast (35.4%), followed by the Northeast (30.6%). However, when comparing the populations of each region in Brazil, the Southeastern region had the lowest number of ULVT cases per inhabitant (despite the higher absolute number of cases), with approximately 10.7 cases per 100,000 inhabitants. The region with the most cases per inhabitant was the North (16.6 cases per 100,000 inhabitants), followed by the Northeast (14.3 per 100,000 inhabitants), Midwest (13.0 cases per 100,000 inhabitants), and South (12.4 cases per 100,000 inhabitants).

ULVT: upper limb vascular trauma.

**Figure 3:**
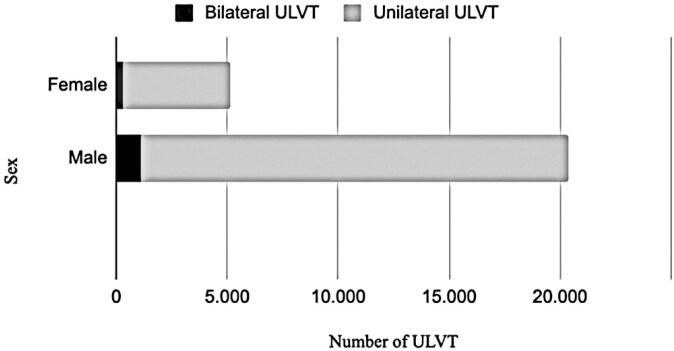
Number of upper limb vascular trauma per sex

Figure 3 shows the distribution of the ULVT according to sex. Males accounted for 79.8% of the cases. The proportions of bilateral and unilateral ULVT in males and females were similar (6.1% and 5.7%, respectively).

ULVT: upper limb vascular trauma.

**Figure 4:**
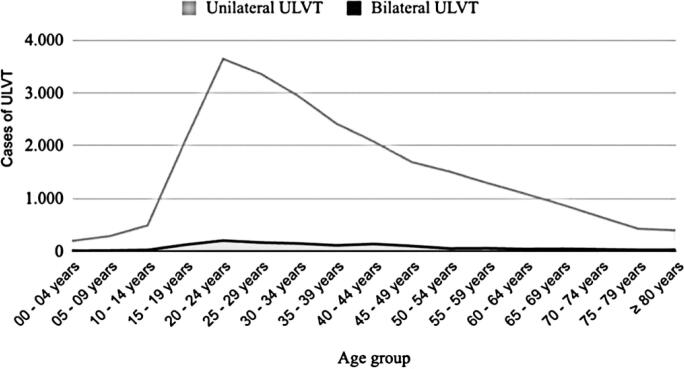
Number of upper limb vascular trauma per age group

The ULVT for each age group is shown in figure 4. There is a peak in the incidence of ULVT at 20 to 24 years of age, with a gradual reduction as age increases. The mean age was 34.7 years.

ULVT: upper limb vascular trauma.

**Figure 5:**
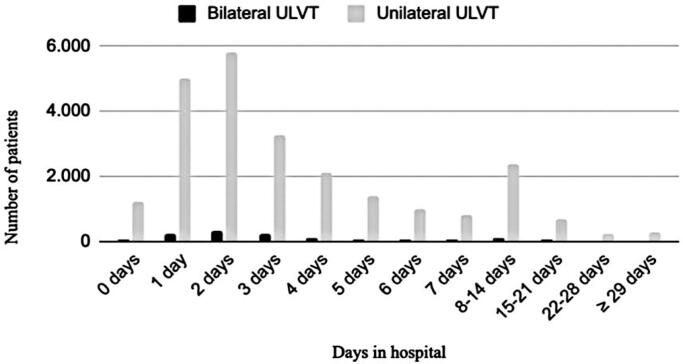
Days of hospitalization for upper limb vascular trauma

Figure 5 shows the number of days of hospitalization. Most patients with ULVT were hospitalized for 2 days, with a gradual decrease in the number of days over time. The average length of hospitalization for patients with ULVT was 4.39 days.

ULVT: upper limb vascular trauma.

**Figure 6:**
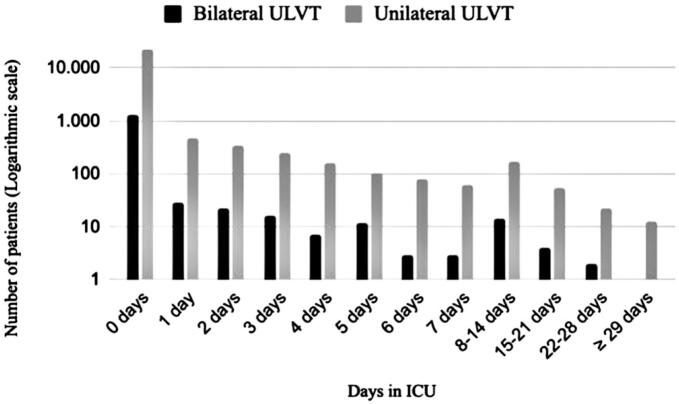
Days in the intensive care unit for patients with upper limb vascular trauma

ULVT: upper limb vascular trauma; ICU: intensive care unit.

Figure 6 shows the length of ICU hospitalization on a logarithmic scale. Most patients (92.8%) did not require ICU admission. In the group of patients who required ICU admission, the average number of days in the ICU was 4.52 days.

**Figure 7:**
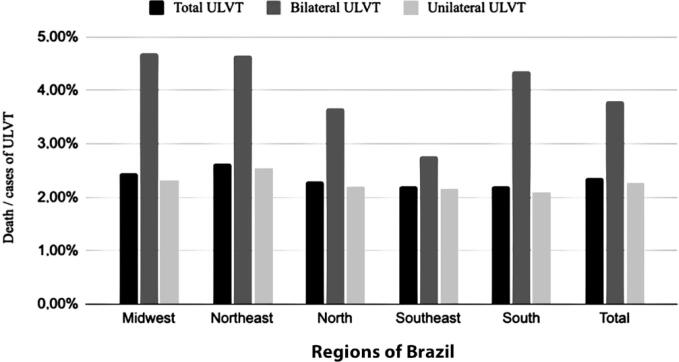
Lethality of upper limb vascular trauma per region of Brazil

The total number of deaths among patients with ULVT was 605, representing a lethality rate (deaths per case) of 2.37%. Lethality in bilateral ULVT was higher than that in unilateral ULVT cases (3.81% and 2.28%, respectively). Figure 7 shows the lethality of unilateral and bilateral ULVT in the five regions of Brazil. The Northeast had the highest lethality rate (2.64%), followed by the Center-West (2.46%). The Southeast region had the lowest lethality for ULVT cases, with no significant difference between bilateral and unilateral trauma (bilateral 2.77% and unilateral 2.17%).

ULVT: upper limb vascular trauma.

**Figure 8:**
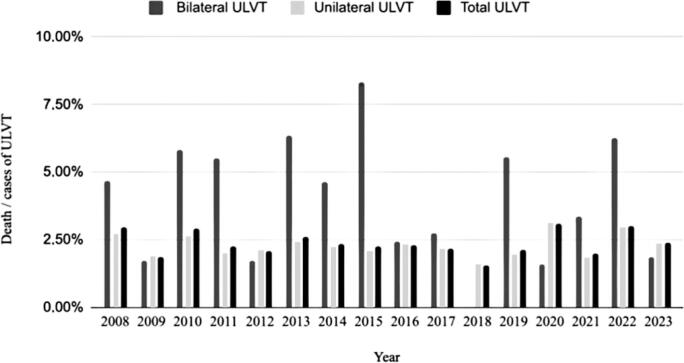
Lethality of upper limb vascular trauma over the years of study

Figure 8 shows the mortality distribution during the study period. Lethality varied considerably over time. The year with the highest number of deaths per ULVT case was 2020, followed by 2008 and 2010.

ULVT: upper limb vascular trauma.

The cost for the public health system to treat all ULVTs from 2008 to 2023 was $8,962,996. Table 1 shows the total expenses by region in Brazil. The Southeastern region spent the most, followed by the Northeastern, which is consistent with the number of ULVTs in these locations.

**Table 1:** Cost of treatment for upper limb vascular trauma in each region of Brazil (in dollars)

	Midwest	Northeast	North	Southeast	South	Total cost	Cost/patient
ULVT	734,387	2,839.172	981,417	3,079,147	1,328,873	8,962,996	350
Bilateral ULVT	41,575	131,461	48,886	181,169	89,311	492,401	354
Unilateral ULVT	691,559	2,704,861	932,531	2,867,163	1,237,669	8,433,783	350

ULVT: upper limb vascular trauma.

**Figure 9:**
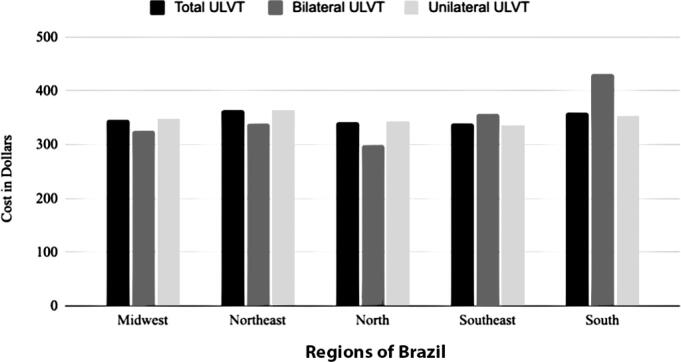
Cost per patient of upper limb vascular trauma (in dollars)

Figure 9 shows the cost per patient of treating total, bilateral, and unilateral ULVT by region in Brazil. The cost per patient did not differ significantly across regions, and it cannot be concluded that treating patients with bilateral injuries was more expensive than treating those with unilateral injuries. The southern region had higher hospitalization costs for patients with bilateral ULVT.

ULVT: upper limb vascular trauma.

## DISCUSSION

Vascular trauma refers to injuries to blood vessels, which can be arterial or venous. These injuries typically result from penetrating or blunt trauma and can occur in various body regions, including the extremities, head, neck, chest, and abdomen. Vascular injuries are more common in males (approximately 80%),^(2)^ with an average age of approximately 35 years.^(3)^ Upper limb vascular trauma is one of the most common types of vascular trauma.^(13)^ Treatments vary, with traditional approaches typically involving open surgery, whereas endovascular methods are becoming more common.^(14,15)^ Epidemiological studies of ULVT involving large populations are scarce, particularly in underdeveloped or developing countries.

The number of ULVT cases in Brazil exceeds those reported in other countries. Despite having a population approximately 50% larger than that of Brazil,^(8,16)^ the United States has reported fewer cases of ULVT. Over five years, Brazil has documented 7,578 cases of ULVT compared to 5,855 cases in the United States. In Scotland, a country with a population 40 times smaller than that of Brazil, 130 ULVT cases were recorded from 2011 to 2018, while Brazil recorded 13.078 ULVT cases during the same period. Proportional to its population size, the incidence of ULVT in Brazil remains higher than that in Scotland. The higher incidence of vascular trauma in Brazil could be attributed to the higher rates of traffic accidents^(17)^ and violence,^(18)^ although further studies are needed to confirm these hypotheses. Regarding the number of ULVTs over time, both unilateral and bilateral trauma cases were stable. The peak period of the SARS-CoV pandemic (2020-2021) did not influence the trend in cases.^(19)^

Notably, during the COVID-19 pandemic, the number of vascular traumas did not decrease, as expected, in the context of lockdown and less exposure to occupational and traffic accidents. A possible explanation could be the increased circulation of motorcycles owing to delivery services in large cities, potentially leading to more traffic accidents. In São Paulo, there was 187% growth in delivery platforms in 2020, according to data from the *Fundação Getúlio Vargas*.^(20)^ In smaller cities, adherence to the lockdown was more limited as people continued going out, which may explain the sustained incidence of upper-limb trauma in these regions. Another explanation could be the increase in domestic accidents during the lockdown, such as those involving home renovations, the use of tools, and other manual activities. According to World Health Organization data, the cases grew by 30% during the pandemic.^(21)^

The number of ULVT cases varied across Brazil. In absolute numbers, the Southeast reported the highest number of cases, followed by the Northeast. However, when adjusted for population size, the Northern region had the highest incidence of ULVT, followed by the Northeastern, Midwestern, Southern, and Southeastern regions. These findings may be attributed to the higher prevalence of traffic accidents in the North (3.4%), followed by the Central-West (3.2%) and Northeast (2.7%), with lower rates observed in the Southeast (2.1%) and South (2.0%).^(22)^ Additionally, physical violence seems to be more prevalent in the Northern and Northeastern regions of Brazil, especially in areas with lower educational attainment and higher firearm availability, which may also contribute to these patterns.^(23)^

The proportion of male patients with ULVT in Brazil was comparable to the global values. In Brazil, 79.8% of ULVT cases occurred in males, whereas in other countries, this number ranged from 68% to 80%. A possible explanation for these findings is that young males are at a higher risk of traffic accidents, which is related to driving behaviors and attitudes such as speeding and alcohol consumption.^(24)^ However, this remains a hypothesis, as the primary cause of ULVT in Brazil has not yet been definitively established. The only study from Latin America, conducted at a single center in Brazil, showed that firearm injuries were the main cause of vascular trauma in all body regions.^(25)^

These data were consistent with age. Patients with ULVT in Brazil had a mean age of 34.7 years, whereas in the six studies, the mean age ranged from 31 to 49 years. This poses a significant issue for the labor market, as it preferentially affects economically active age groups, thereby reducing employment prospects and monthly earnings over time. Accidents, violence, and chronic diseases are among the leading causes of economic loss.^(26)^

The mean length of hospital stay for patients with ULVT varied little among the countries studied. In the United States, two studies reported average stays of 4.6 and 5.7 days,^(1,6)^ whereas in Australia, one study reported an average stay of two days.^(4)^ Meanwhile, in Brazil, the mean length of hospital stay for patients with ULVT was 4.39 days. Therefore, the duration of hospital stay was relatively consistent across the countries studied.

Two studies from the United States reported an average ICU stay of 4.5 and 4.6 days for patients with ULVT.^(1,6)^ These results are consistent with those of Brazil, where the average length of ICU stay was 4.52 days, although only 7.2% of patients with ULVT required intensive treatment.

The lethality of patients with ULVT in Brazil was 2.37%, compared to 2.2–2.9% in the United States,^(1,6)^ 9% in Scotland,^(3)^ and 0.9% in Australia.^(4)^ Within Brazil, the Northeast region had the highest lethality rate for patients with ULVT, followed by the Midwest, North, South, and Southeast regions. One hypothesis for this difference is the uneven distribution of the country's largest medical centers, which are more concentrated in the Southeast and less so in the North.^(27)^ With regard to mortality from trauma (bilateral or unilateral), higher rates were observed for bilateral trauma for most of the study period, which may be explained by its association with thoracic trauma or by the greater severity of the condition, as in the case of polytraumatized patients. However, the DATASUS database filters information using a procedure code, which makes it difficult to analyze each case specifically.

The disparity between Brazilian data and those of developed countries can also be explained by occupational safety policies. Despite the rules for implementing the use of personal protective equipment established in NR 36, employees in Brazil reluctantly use these devices, an issue worsened by the negligence and permissiveness of employers, who often fail to provide adequate training and guidance.^(28)^

To date, no studies on large populations have assessed the cost of ULVT in healthcare systems. This study observed that a total of $8,962,996 was spent on the acute treatment of these injuries, representing a cost of $350 per patient. However, additional costs for post-hospitalization therapies such as rehabilitation, follow-up consultations, and work incapacity were not considered. These costs can be much higher when considering that these are generally injuries in economically active populations who may partially or completely lose their ability to work. The cost referred to in the results was estimated based on the amount transferred by the government to the Unified Health System and data extracted from the DATASUS website, which may underestimate the real cost of hospitalization and complementary procedures to which the patient underwent treatment and recovery from traumatic injuries. However, no other method is available to accurately determine the cost of this diagnosis in a nationwide sample.

Upper limb vascular trauma is more prevalent in Brazil than in other countries, even after adjusting for population size. This suggests that underdeveloped and developing countries may experience higher upper limb vascular trauma rates than developed countries. However, the lethality and length of hospitalization for upper limb vascular trauma in Brazil did not exceed those observed in developed countries. There are some regional variations in the incidence and lethality of upper limb vascular trauma in Brazil.

### Limitations

This study has a few limitations. First, the exact sample size could not be determined. The sample comprised at least 140 million people, as this is the number of people who rely exclusively on the SUS. However, this number is likely higher as trauma is usually treated acutely by the SUS, even if the patient is later transferred to the private system. Thus, the study population varied from approximately 140 to 200 million people.

Second, the possibility of measurement bias exists, which is common in population-based studies. This could occur because the study relied on the medical records documented by healthcare professionals, which may underestimate the number of vascular traumas, particularly those involving other traumas.

A study evaluating the recording of hospitalization data in private hospitals in Rio de Janeiro showed that, despite low reliability in recording clinical diagnoses, the documentation of surgical procedures was highly reliable.^(29)^ This reiterates the validity of our data, which were filtered by the sympathectomy code. Additionally, another study carried out in public hospitals in Paraná showed low accuracy in recording diagnoses,^(30)^ likely due to inadequate training and a lack of knowledge of the correct codes by the professionals. These findings highlight the need for better orientation and awareness programs to improve data integrity.

Another limitation is that we did not account for medically treated ULVTs or primary amputations. The data obtained were extracted using surgical codes, which limited access to the number of clinical treatments and may have underestimated the number of ULVTs. Nevertheless, limb trauma is mostly treated surgically,^(31,32)^ which allows this number to be extrapolated to the total number of ULVTs for analysis. In practice, clinically treated vascular injuries are typically not recorded as vascular injuries. Moreover, other studies reviewed did not distinguish between clinical and surgical treatments.

Furthermore, the costs presented in this study are the reimbursements paid to the SUS based on a fixed schedule; therefore, they may not accurately reflect the actual costs to the hospital.

## CONCLUSION

Upper limb vascular trauma is more prevalent in Brazil than in developed countries and primarily affects young males. Although regional variations in the incidence and mortality exist, these differences were not significantly pronounced. This study underscores the need for targeted prevention strategies and calls for further research on the causes and economic impacts of upper limb vascular trauma in developing countries.
